# Application of a new position in endoscopic combined intrarenal surgery: modified prone split-leg position

**DOI:** 10.1186/s12894-022-00994-w

**Published:** 2022-03-19

**Authors:** Daming Wang, Hongliang Sun, Dongdong Xie, Zhiqi Liu, Dexin Yu, Demao Ding

**Affiliations:** 1 Department of Urology, The Second Affiliated Hospital of Anhui Medical University, Hefei, 230601 China; 2 Department of Urology, Taikang Xianlin Drum Tower Hospital, Nanjing University, Nanjing, 210000 China

**Keywords:** Endoscopic combined intrarenal surgery, Percutaneous nephrolithotomy, Retrograde intrarenal surgery, Galdakao-modified supine Valdivia, Prone split-leg position

## Abstract

**Background:**

Endoscopic combined intrarenal surgery (ECIRS) is well established as a minimally invasive procedure for the treatment of multiple urolithiasis. The position is the key to the perfect combination of percutaneous nephrolithotomy (PCNL) and retrograde intrarenal surgery (RIRS). Galdakao-modified supine Valdivia (GMSV) and prone split-leg positions are widely used. However, both positions have their own advantages and disadvantages. This study aimed to evaluate the effect of ECIRS in the treatment of multiple urolithiasis in the modified prone split-leg position.

**Patients and methods:**

A total of 96 patients with multiple urolithiasis underwent ECIRS in modified prone split-leg position from September 2017 to January 2021. Relevant demographic and clinical data were analysed retrospectively. Clinical outcomes, such as the stone free rate, complications and postoperative hospital stay were evaluated. The chi-square test was used to compare categorical variables and Student’s t test was applied for continuous variables of the treatment groups.

**Results:**

The mean renal stone size was 32.5 ± 10.7 mm and renal stone surface area was 712.2 ± 264.8 mm^2^. The mean ureteral stones size was 24.8 ± 12.3 mm. The mean surgical time was 82.2 ± 38.3 min. The incidence of complications was 16.7%, and they were mainly grade 1 and grade 2. No complications occurred above grade 3. The stone was completely removed in 75 (78.1%) patients in a single operation. The risk factors affecting the stone-free rate of ECIRS were analysed, and only the number of involved calyces by stone was found to be significant (*p* = 0.01).

**Conclusion:**

ECIRS is safe and effective in the treatment of multiple renal calculi or multiple renal calculi with ipsilateral ureteral calculi in the modified prone split-leg position. The modification of the prone split-leg position makes the retrograde operation more convenient, which is conducive to the combination of RIRS and PCNL.

## Background

Endoscopic combined intrarenal surgery (ECIRS) is a minimally invasive method that combines percutaneous nephrolithotomy (PCNL) and retrograde intrarenal surgery (RIRS) for the treatment of complex upper urinary tract calculi. This combination can reduce the number of operations and puncture channels for the treatment of complex stones [1, 2]. In addition, it can increase the stone clearance rate in a single operation [3]. This position is the key to the perfect combination of PCNL and RIRS. The Galdakao-modified supine Valdivia (GMSV) position is gaining popularity worldwide [1, 2, 4, 5]. Its advantage is that it facilitates anaesthesia management and has no significant effect on the respiratory and circulatory systems [1]. The disadvantage of this position is that the puncture space is limited, especially the upper calex puncture, and the risk of visceral injury is high [6]. The prone position is common in percutaneous nephrolithotomy and urologists are familiar with it, and the prone split-leg position is also widely used in ECIRS [7, 8]. However, during an actual operation in the prone split-leg position, the lower limbs on the nonoperative side of the patient had greater interference with the retrograde operation of the ureteroscope, especially for male patients. Therefore, we modified the prone split-leg position. This study aimed to evaluate the effect of ECIRS in the treatment of multiple renal calculi or multiple renal calculi with ipsilateral ureteral calculi in the modified prone split-leg position.

## Patients and methods

Between September 2017 and May 2020, 96 patients who underwent ECIRS in the modified prone split-leg position at the Second Affiliated Hospital of Anhui Medical University were retrospectively reviewed and analysed. The study was approved by the Research Ethics Committee at Second Affiliated of Anhui Medical University. All experiments were performed in accordance with the EAU (European Association of Urology) and AUA (American Urological Association) guidelines. Patient demographics are displayed in Table 1. The preoperative patient evaluation included the history, clinical examination, routine laboratory investigations (basal parameters and urine bacterial culture), and anesthesiology risk evaluation (American Society of Anesthesiologists) risk before general anesthesia. The location, size, and density of the stones were evaluated by preoperative imaging studies. All patients had an unenhanced computed tomography (CT) scan and a plain film of the abdomen. Stone size was determined by measuring the longest diameter on preoperative radiologic investigations; in the case of multiple calculi, it was defined as the sum of the longest diameter of each stone. The stone surface area was estimated using the formula described by Tiselius and Andersson (length × width × 3.14 × 0.25) [9]. Urine bacterial culture was completed in all patients after admission. According to the situation of urinary tract infection, reasonable antibiotics were selected before the operation. There were no patients with pre placed stents in our study.

**Table 1:** Patient and stone demographics

Characteristic	Value
Characteristics of patients and stone features, mean ± SD	
Age (years)	55.4 ± 10.5
N (male:female)	51/45
BMI (kg/m^2^)	24.3 ± 3.1
Hb (g/dL)	12.7 ± 1.9
Hct (%)	38.6 ± 5.3
Side (right:left)	54/42
ASA status, n (%)	
ASA I	44 (45.8)
ASA II	45 (46.9)
ASA III	7 (7.3)
Multiple renal calculi, n (%)	61 (63.5)
Multiple renal calculi with ipsilateral ureteral calculi, n (%)	35 (36.5)
Renal stone size (mm)	32.5 ± 10.7
Renal stone surface area (mm^2^)	712.2 ± 264.8
Number of calyces involved by stone, n (%)	
< 3	42 (43.8)
≥ 3	54 (56.2)
CT (HU)	1055.9 ± 301.1
Ureteric stone size (mm)	24.8 ± 12.3
Ureteric stone number	3.2 ± 1.2
Preoperative history, n	
SWL	12
URS	9
PCNL	14
Operative parameters, mean ± SD	
Surgical time (min)	82.2 ± 38.3
Hb drop (g/dL)	0.9 ± 0.5
Hct drop (%)	2.7 ± 1.1
Postoperative hospital stay (d)	6.5 ± 1.3
Stone free, n (%)	75(78.1)
Ancillary treatment	
Total	11
Second PCNL	2
SWL	6
FURSL	3
Intra and postoperative complications according to the	
modified Clavien classification	
Clavien grade 0, n (%)	80 (83.3)
Clavien grade I, n (%)	14 (14.6)
Transient fever > 38.5℃	9 (9.4)
Hemorrhage, non transfusion	5 (5.2)
Clavien grade II, n (%)	2 (2.1)
Blood transfusion	2 (2.1)
Clavien grade ≥ III, n (%)	0 (0)

*Hb* hemoglobin, *Hct* Hematocrit, *BMI* body mass index, *ASA* American Society of Anesthesiologists, *CT* computed tomography, *HU* hounsfield unit, *SWL* shock wave lithotripsy, *PCNL* Percutaneous nephrolithotomy, *FURSL* flexible ureteroscope

### Surgical techniques

**Fig. 1:**
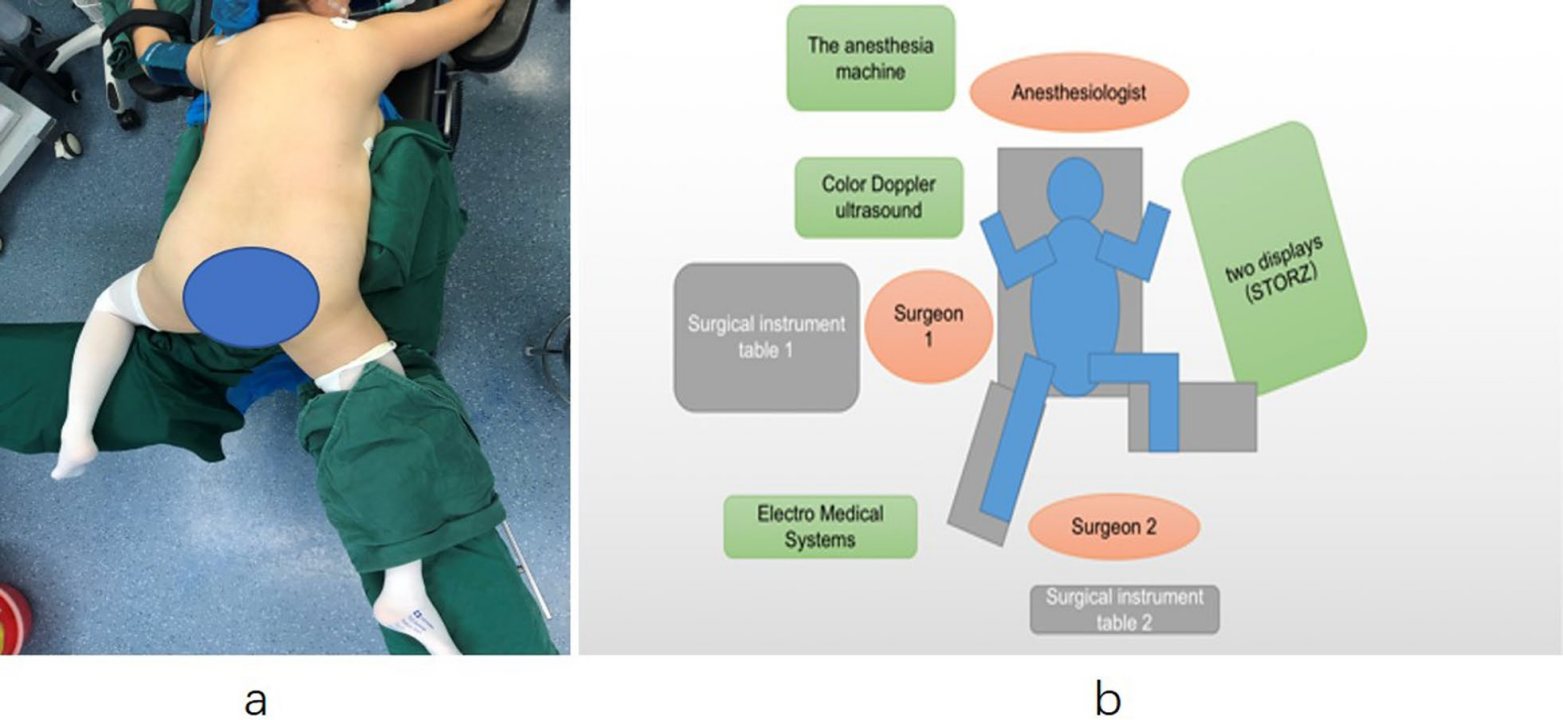
**a** The patient was placed in a modified prone split-leg position with the treatment side on the right. **b** The schematic diagram shows the position of the patient in the modified prone-split leg position with the treatment side on the left, the position of the surgeon and the placement of the instruments

**Fig. 2:**
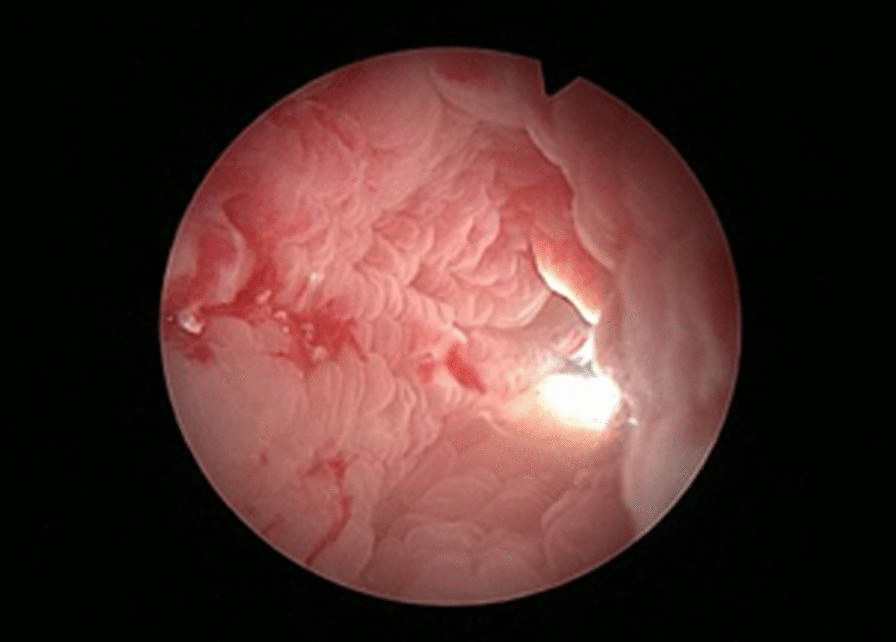
Ureteroscopy can enter the renal pelvis and meet with the nephroscope

**Fig. 3:**
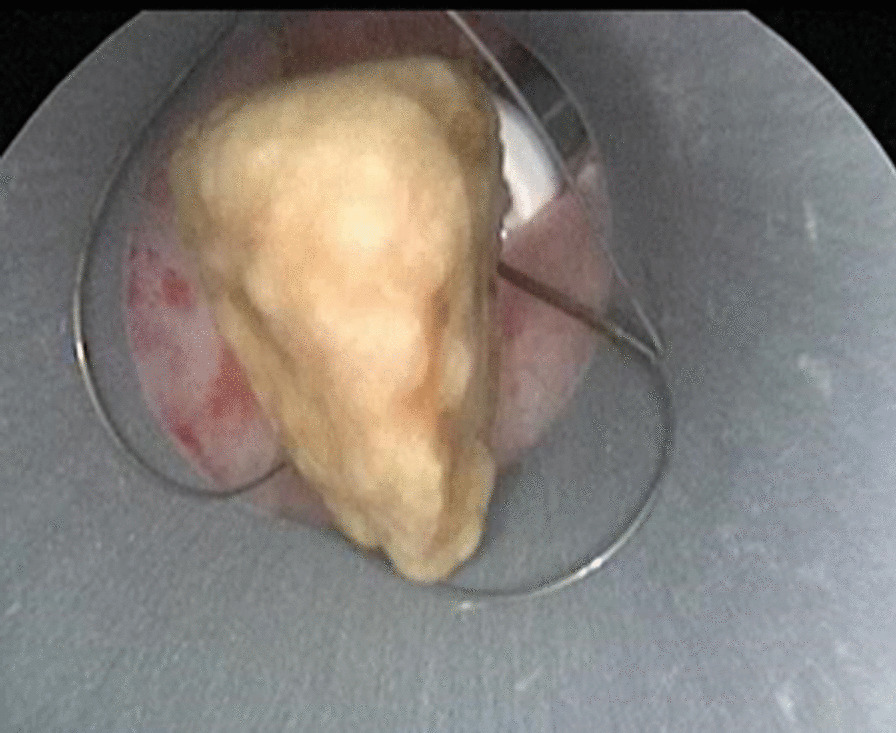
Flexible ureteroscopy assisted percutaneous nephrolithotomy for the removal of stones that cannot be detected by nephroscopy

**Fig. 4:**
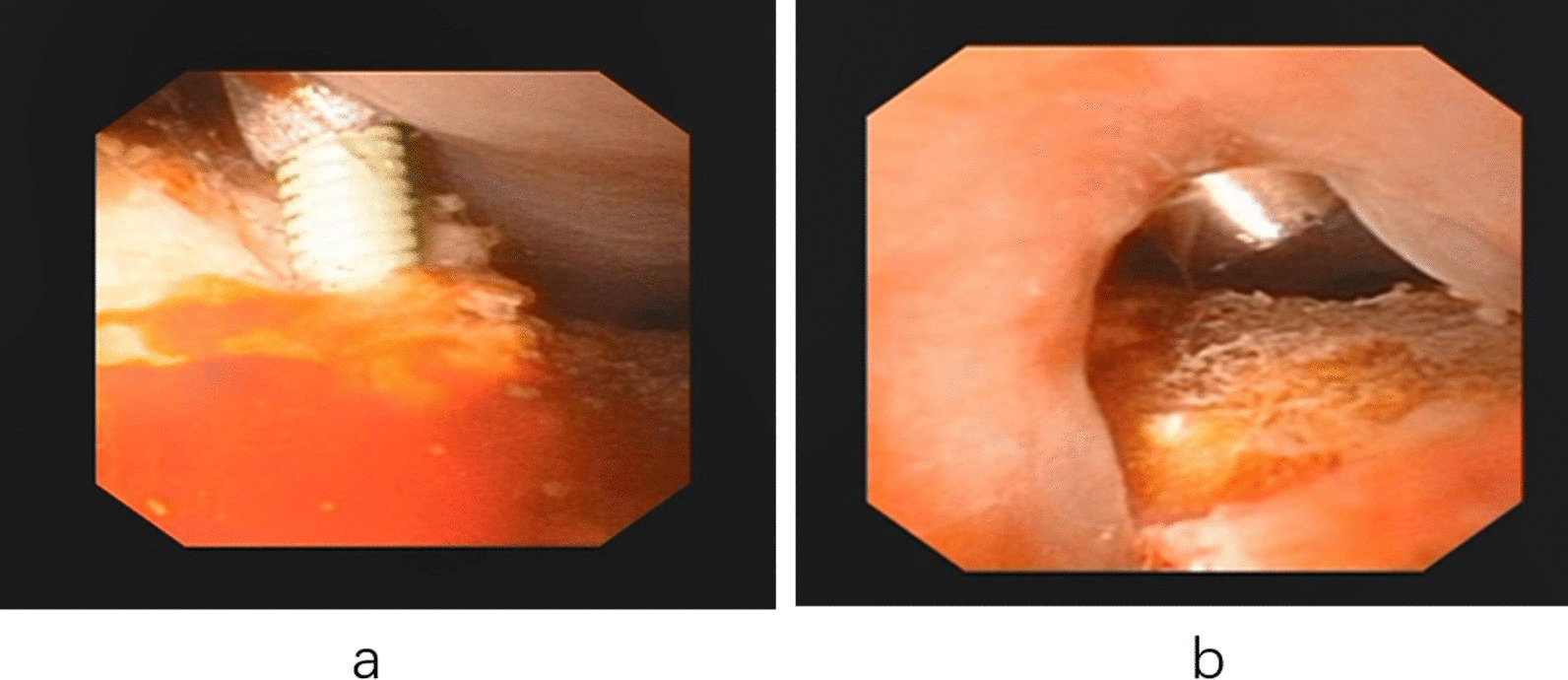
**a** The position of the puncture needle was monitored by flexible ureteroscope. **b** The establishment of the puncture channel was monitored by flexible ureteroscope

All patients were given general anaesthesia. The patients were treated with a new posture: modified prone split-leg position. The patients were placed in the prone position, and silicone pads were placed under the face and chest to avoid compression of the eyes and tracheal intubation. The tip of twelfth rib was used as the midpoint, and the sponge pad was placed under the abdomen. The legs were then placed on the leg board. On the nonoperative side, the leg plate was expanded to 90°, hip joint flexion was approximately 90°, and abduction and knee flexion were 90°. The lower limb of the operative side was straight and abducted approximately 15°. The operation was performed by two urologists at the same time, with one performing PCNL and the other performing transurethral retrograde surgery (Fig. 1a, b). All patients in our study were operated by the same two urologists. First, the head side of the operating table was lowered to form an angle of 30° with the horizontal line. Retrograde transurethral access to ureteroscopy (Karl Storz, Tuttlingen, Germany) was performed by surgeon 2. It is easier to enter the bladder in female patients than male patients; therefore, an F12 catheter can be placed first, and the bladder can enter along the catheter. The ureteral orifice was located at 11 o'clock (left side) and 1 o'clock (right side). The ureteroscope enters the ureter under the guidance of a guide wire. Pneumatic lithotripsy (Electro Medical Systems, Swiss LithoClast® Maste, Nyon, Switzerland) was performed for lower ureteral calculi. Additionally, ultrasound-guided percutaneous nephroscopy was performed by surgeon 1. The standard F22/24 channel was established, and ultrasonic (ultrasonic energy 80%, duty cycle 70%, Electro Medical Systems, Swiss LithoClast® Maste, Nyon, Switzerland) or pneumatic ballistic lithotripsy was used under the supervision of a nephroscope (f20, Wolf, Germany). If the F14-18 channel was established, then a holmium:yttrium–aluminium-garnet (YAG) laser was used. The upper ureteral calculi were pushed into the renal pelvis by surgeon 2 and removed through the sheath after lithotripsy by surgeon 1. Ureteroscopy can enter the renal pelvis and meet with the nephroscope (Fig. 2). If there were multiple kidney stones or stones that could not be found by nephroscopy, the F12/14 flexible ureteroscope sheath was placed retrogradely by surgeon 2, and the flexible ureteroscope (Flex X-2, Karl Storz, Tuttlingen, Germany) was retrogradely inserted into the renal pelvis to help the nephroscope find stones or use holmium laser lithotripsy (Fig. 3). In addition, the establishment of a percutaneous nephroscope channel could be monitored (Fig. 4a, b). Finally, the ureteral stent was placed anterograde and ECIRS was used to ensure the correct position of the stent tube. The ureteral stent was removed one month later.

### Postoperative evaluation

On the day after surgery, the laboratory examination, including measurements of haemoglobin and haematocrit levels were compared with those before operation. CT was reexamined 1–3 months after the operation to evaluate the effect of stone removal. Stone-free status was defined as the stone being completely removed or the stone fragment being less than 2 mm [10]. Complications were graded according to the modified Clavien classification [11]. All patients were examined by stone composition analysis after operation.

### Statistical methods

Continuous variables were summarized as the mean ± SD. Categorical variables are represented by frequencies and percentages. The chi-square test was used to compare categorical variables and Student’s *t* test was applied for continuous variables of the treatment groups. *p* < 0.05 indicated significant findings.

## Results

### Stone features

The characteristics of the stones are summarized in Table 1. Thirty-five patients (36.5%) had multiple renal calculi with ipsilateral ureteral calculi and 61 (63.5%) had multiple renal calculi. The mean renal stone size was 32.5 ± 10.7 mm and the renal stone surface area was 712.2 ± 264.8 mm^2^. The mean radiodensity of the stones was 1055.9 ± 301.1 HU. The mean ureteral stone size was 24.8 ± 12.3 mm.

### Surgical data

All patients underwent single tract percutaneous nephrolithotomy.

The mean surgical time was 82.2 ± 38.3 min. The mean decrease in Hb level was 0.9 ± 0.5 g/dL, and the mean decrease in Hct level was 2.7 ± 1.1%. The mean postoperative hospital stay was 6.5 ± 1.3 days (Table 1).

### Stone-free rate

At the end of the first intervention, all patients achieved complete clearance with respect to ureteric calculus. The stone was completely removed in 75 (78.1%) patients in a single operation. Eleven patients required reoperation: 6 SWL, 3 fURS, and 2 PCNL (Table 1). The risk factors affecting the stone free rate of ECIRS were analyzed, and only the number of involved calyces by stone was found to be significant (*p* = 0.01) (Table 2).

**Table 2:** Risk factors for residual stones

Variables	SF (n = 75)	Non-SF (n = 21)	*p*
Age (years)	56.1 ± 9.1	52.8 ± 14.8	0.51
BMI (kg/m^2^)	24.2 ± 3.4	24.6 ± 1.7	0.69
Sex			
Male	41	10	0.57
Female	34	11	
Side			
Right	43	11	0.69
Left	32	10	
Renal stone size (mm)	31.8 ± 11.1	35.1 ± 9.3	0.39
Renal stone surface area (mm^2^)	677.1 ± 261.8	838.7 ± 247.6	0.09
CT (HU)	1064.4 ± 302.6	1025.2 ± 309.9	0.72
Stone analysis			
Calcium oxalate	54	15	0.96
Non calcium oxalate	21	6	
Number of calyces involved by stone			
< 3	38	4	0.01
≥ 3	37	17	

*BMI* body mass index, *CT* computed tomography, *HU* hounsfield unit, *SF* stone free

### Complications

The complications are summarized in Table 1. Nine patients had transient fever on the first day after the operation. The symptoms improved after treatment with broad-spectrum antibiotics and antipyretic drugs. Five patients suffered from haemorrhage without blood transfusion and improved after conservative treatment. Two cases of massive haemorrhage required blood transfusion. None of the patients had Clavien grading scores ≥ 3. The total complication rate was 16.7%.

## Discussion

The EAU and AUA guidelines recommend PCNL as the first-line choice for the treatment of large, staghorn and multiple renal calculi [12, 13]. However, for complex renal calculi such as staghorn calculi and multiple renal calculi, a large or multichannel approach is needed to improve the stone clearance rate. In a single channel, the nephroscope attempts to reach each calyx, which could damage the calyx neck and haemorrhage, thereby increasing the rate of blood transfusion and urinary extravasation [14]. Multichannel percutaneous nephroscopy is associated with large renal parenchymal injury [15]. In addition, 29–36% of complex renal calculi are complicated with multiple ipsilateral ureteral stones [4]. Traditional surgical treatment requires staged lithotripsy or ureteroscopic lithotripsy followed by changing the body position for PCNL. When RIRS is used alone, the pressure in the renal pelvis will be increased, which could lead to the extravasation of perfusion fluid and infection. In addition, in the process of changing the body position or lithotripsy, the stone could migrate into the ureter, which could cause difficulty in lithotripsy, and even the body position is changed again. How to combine PCNL with RIRS in one position is the key issue when dealing with complex renal calculi. At present, the GMSV position and prone split-leg position are widely used, and good results have been achieved. The GMSV position is close to the daily physiological position of patients and has little influence on cardiovascular and respiratory movement after general anaesthesia, especially in obese patients [16]. However, due to the influence of gravity, the continuous perfusion pressure decreases during PCNL, and the renal collecting system cannot be filled. The aggregation of bubbles will affect the clarity of vision and gravel. In addition, the space of renal puncture is limited, and the difficulty is increased, especially in the upper calices, which will increase the risk of visceral injury [17, 18]. The space of puncture in the prone split-leg position is large, and most urologists are familiar with percutaneous nephrolithotomy in the prone position. In the prone position, the upper ureter and kidney move to the ventral side due to gravity, which leads to straight ureteral passage and reduces the tortuosity and angle. Performing retrograde ureterorenoscopy of the proximal ureter and renal pelvis is easier [7]. Under the action of gravity and water pressure, the upper ureteral calculi and the dorsal calyceal calculi can be concentrated at the lower part of the renal pelvis, which is conducive to looking for stones under nephroscopy. The prone position is also related to the depth of the puncture channel, which can be reduced, and more puncture sites can be provided, which can reduce the difficulty of puncture and improve the safety of PCNL [6]. However, during the retrograde ureteroscopy operation, the interference of the contralateral lower limb was greater. Because in the prone split-leg position, the angle of separation of the two lower limbs is approximately 60–80 degrees. During the retrograde ureteroscopy operation, the body of the extracorporeal mirror moves to the opposite side, and the interference of the contralateral lower limb is greater. The actual operating area is only half. In the modified prone split-leg position, the hip and knee flexion of the nonoperative side was 90 degrees. The abduction angle of the lower limb on one side of the operation is small, which avoids excessive stretching of the thigh muscles. The actual operation space was significantly increased, and interference of the contralateral lower limb on retrograde operation was avoided. In the modified position, the lower limb muscles of the patient are completely relaxed, and the limb flexion angle is within the normal range. All patients had no nerve injury or sensory/motor complaints. The learning curve of ureteroscopy in prone position is short. In addition, there was no ureteral injury caused by ureteroscopy in our study.

Our study demonstrates that the initial renal stone-free rate was 78.1%, and the ureteral calculi were completely removed; moreover, only 11.5% of patients required secondary treatment. Manikandan et al. also demonstrated a similar success for the management of complex renal and ureteric stones with 18% of patients requiring secondary treatment [4]. Hamamoto et al. showed that the renal stone free rate of ECIRS in the prone split-leg position was 71.4% [8]. Most reports indicate that the renal stone-free rate of ECIRS in the GMSV position is 65.3–87.88% [5, 19–21]. Hamamoto et al. showed that the stone size, stone surface area, complete staghorn calculi, and number of stone branches were risk factors for residual stones in ECIRS [8]. Manikandan et al. reported that only the number of calyces involved by stones was significantly associated with the stone-free rate after ECIRS [4], and our study is consistent with this report. However, Yamashita et al. reported that stone size was a risk factor for residual stones, and the number of involved calyces was not predictive [22].

The incidence of complications in our study was 16.7%, which was significantly lower than that of PCNL reported in the literature [23, 24]. Manikandan et al. reported that the complication rate of ECIRS in the GMSV position was 32.5% [4]. There were no grade 3 or above complications in our study. ECIRS has obvious advantages in reducing complications. Ureteroscopy can monitor the puncture and expansion, determine the puncture site and avoid injury. Stones located in the parallel calices of the puncture channel or stones that cannot be found by the nephroscope can be moved by a retrograde flexible ureteroscope; in addition, direct lithotripsy can be performed to avoid the risk of bleeding caused by excessive swing of the nephroscope. The two kinds of endoscopes were operated simultaneously, thus providing mutual drainage channels to reduce the pressure in the renal pelvis and prevent infection. In addition, the upper and lower channels can keep the drainage unobstructed and avoid high pressure in the renal pelvis and ureter, which can not only keep the visual field clear but also reduce the incidence of infection. Although a comparative study was not performed, ECIRS in the modified position does not prolong the postoperative hospital stay compared with ordinary PCNL.

The development of instruments and display technology, such as Storz split screen display and disposable flexible ureteroscope, reduces the occupation and cost of equipment, which is more conducive to the development of this technology. The limitation of this study is that it was a retrospective study with a small sample size and a descriptive rather than a comparative study. In future studies, we will increase the sample size and evaluate the selection criteria of this technology.

## Conclusion

ECIRS is safe and effective in the treatment of multiple renal calculi or multiple renal calculi with ipsilateral ureteral calculi in the modified prone split-leg position. It can be used to avoid repeated changes in body position, shorten operation time, improve stone clearance rate and reduce complications. The modification of the prone split-leg position makes the retrograde operation more convenient, which is conducive to the combination of RIRS and PCNL.

## Data Availability

The datasets used during this study available from the corresponding author on reasonable request.

## References

[1] Scoffone CM, Cracco CM, Cossu M, Grande S, Poggio M, Scarpa RM (2008). Endoscopic combined intrarenal surgery in galdakao-modified supine valdivia position: a new standard for percutaneous nephrolithotomy?. Eur Urol.

[2] Cracco CM, Scoffone CM (2011). ECIRS (Endoscopic Combined IntraRenal Surgery) in the Galdakao-modified supine Valdivia position: a new life for percutaneous surgery?. World J Urol.

[3] Hamamoto S, Yasui T, Okada A, Taguchi K, Kawai N, Ando R, Mizuno K, Kubota Y, Kamiya H, Tozawa K (2014). Endoscopic combined intrarenal surgery for large calculi: simultaneous use of flexible ureteroscopy and mini-percutaneous nephrolithotomy overcomes the disadvantageous of percutaneous nephrolithotomy monotherapy. J Endourol.

[4] Manikandan R, Mittal JK, Dorairajan LN, Mishra AK, Sreerag KS, Verma A (2016). Endoscopic Combined intrarenal surgery for simultaneous renal and ureteral stones: a retrospective study. J Endourol.

[5] Wen J, Xu G, Du C, Wang B (2016). Minimally invasive percutaneous nephrolithotomy versus endoscopic combined intrarenal surgery with flexible ureteroscope for partial staghorn calculi: a randomised controlled trial. Int J Surg.

[6] Duty B, Waingankar N, Okhunov Z, Ben Levi E, Smith A, Okeke Z (2012). Anatomical variation between the prone, supine, and supine oblique positions on computed tomography: implications for percutaneous nephrolithotomy access. Urology.

[7] Hamamoto S, Yasui T, Okada A, Takeuchi M, Taguchi K, Shibamoto Y, Iwase Y, Kawai N, Tozawa K, Kohri K (2014). Developments in the technique of endoscopic combined intrarenal surgery in the prone split-leg position. Urology.

[8] Hamamoto S, Yasui T, Okada A, Koiwa S, Taguchi K, Itoh Y, Kawai N, Hashimoto Y, Tozawa K, Kohri K (2015). Efficacy of endoscopic combined intrarenal surgery in the prone split-leg position for staghorn calculi. J Endourol.

[9] Tiselius HG, Andersson A (2003). Stone burden in an average Swedish population of stone formers requiring active stone removal: how can the stone size be estimated in the clinical routine?. Eur Urol.

[10] Schoenthaler M, Wilhelm K, Katzenwadel A, Ardelt P, Wetterauer U, Traxer O, Miernik A (2012). Retrograde intrarenal surgery in treatment of nephrolithiasis: is a 100% stone-free rate achievable?. J Endourol.

[11] Dindo D, Demartines N, Clavien P (2004). Classification of surgical complications. Ann Surg.

[12] Türk C, Petřík A, Sarica K, Seitz C, Skolarikos A, Straub M, Knoll T (2016). EAU guidelines on interventional treatment for urolithiasis. Eur Urol.

[13] Assimos D, Krambeck A, Miller NL, Monga M, Murad MH, Nelson CP, Pace KT, Pais VM, Pearle MS, Preminger GM (2016). Surgical management of stones: american urological association/endourological society guideline. Part I. J Urol.

[14] Aron M, Yadav R, Goel R, Kolla SB, Gautam G, Hemal AK, Gupta NP (2005). Multi-tract percutaneous nephrolithotomy for large complete staghorn calculi. Urol Int.

[15] El-Nahas AR, Shokeir AA, El-Assmy AM, Mohsen T, Shoma AM, Eraky I, El-Kenawy MR, El-Kappany HA (2007). Post-percutaneous nephrolithotomy extensive hemorrhage: a study of risk factors. J Urol.

[16] Ibarluzea G, Scoffone CM, Cracco CM, Poggio M, Porpiglia F, Terrone C, Astobieta A, Camargo I, Gamarra M, Tempia A (2007). Supine Valdivia and modified lithotomy position for simultaneous anterograde and retrograde endourological access. BJU Int.

[17] Sohail N, Albodour A, Abdelrahman KM (2017). Percutaneous nephrolithotomy in complete supine flank-free position in comparison to prone position: a single-centre experience. Arab J Urol.

[18] Daels F, Gonzalez MS, Freire FG, Jurado A, Damia O (2009). Percutaneous lithotripsy in Valdivia-Galdakao decubitus position: our experience. J Endourol.

[19] Kuroda S, Ito H, Sakamaki K, Tabei T, Kawahara T, Terao H, Fujikawa A, Makiyama K, Yao M, Matsuzaki J (2015). Development and internal validation of a classification system for predicting success rates after endoscopic combined intrarenal surgery in the modified Valdivia position for large renal stones. Urology.

[20] Inoue T, Kinoshita H, Okada S, Hamamoto S, Taguchi M, Murota T, Matsuda T (2016). Wideband Doppler ultrasound-guided mini-endoscopic combined intrarenal surgery as an effective and safe procedure for management of large renal stones: a preliminary report. Urology.

[21] Jung HD, Kim JC, Ahn HK, Kwon JH, Han K, Han WK, Kim MD, Lee JY (2018). Real-time simultaneous endoscopic combined intrarenal surgery with intermediate-supine position: washout mechanism and transport technique. Investig Clin Urol.

[22] Yamashita S, Kohjimoto Y, Iba A, Kikkawa K, Hara I (2017). Stone size is a predictor for residual stone and multiple procedures of endoscopic combined intrarenal surgery. Scand J Urol.

[23] de la Rosette J, Assimos D, Desai M, Gutierrez J, Lingeman J, Scarpa R, Tefekli A (2011). The clinical research office of the endourological society percutaneous nephrolithotomy global study: indications, complications, and outcomes in 5803 patients. J Endourol.

[24] Koras O, Bozkurt IH, Yonguc T, Degirmenci T, Arslan B, Gunlusoy B, Aydogdu O, Minareci S (2015). Risk factors for postoperative infectious complications following percutaneous nephrolithotomy: a prospective clinical study. Urolithiasis.

